# Supplementary material for: Novel Acetylcholinesterase Inhibitors Based on Uracil Moiety for Possible Treatment of Alzheimer Disease
Source: Molecules. 2020 Sep 12;25(18):4191. doi: 10.3390/molecules25184191 (PMC7571187; doi:10.3390/molecules25184191)
Supplement: Supplementary file 1 [file molecules-25-04191-s001.pdf]

The Supplementary Data

Novel acetylcholinesterase inhibitors based on uracil moiety for possible treatment of Alzheimer's disease

Vyacheslav E. Semenov [1,*], Irina V. Zueva [1], Marat A. Mukhamedyarov [2], Sofya V. Lushchekina [3], Elena O. Petukhova [2], Lilya M. Gubaidullina [1], Evgeniya S. Krylova [1], Lilya F. Saifina [1], Oksana A. Lenina [1], Konstantin A. Petrov [1,4*]

[1] Arbuzov Institute of Organic and Physical Chemistry, FRC Kazan Scientific Center of RAS, Arbuzov str. 8, Kazan 420088, Russia; zueva.irina.vladimirovna@gmail.com (I.V.Z.); salyakhova.lm@mail.ru (L.M.G.); evgeniya.s.krylova@yandex.ru (E.S.K.); dimple@mail.ru (L.F.S.); leninaox@mail.ru (O.A.L.)

[2] Kazan State Medical University, Kazan 420012, Russia; maratm80@list.ru (M.A.M.); petukhovaeo@mail.ru (E.O.P.)

[3] Emanuel Institute of Biochemical Physics, Kosygina st. 4, Moscow, 119334, Russia; sofya.lushchekina@gmail.com (S.V.L.)

[4] Kazan Federal University, Kremlyovskaya str., 18, Kazan, 420008, Russia

Table of contents

Pages

NMR study of the compounds synthesized……S2

NMR experiments details……S2

MS experiments details……S2

Figure S1. 1D ^{1}H NMR spectrum of **9a** in $CDCl_3$ at T = 303 ……S3

Figure S2. 1D ^{13}C NMR spectrum of **9a** in $CDCl_3$ at T = 303……S3

Figure S3. 1D ^{1}H NMR spectrum of **10b** in $CDCl_3$ at T = 303……S4

Figure S4. 1D ^{13}C NMR spectrum of **10b** in $CDCl_3$ at T = 303……S4

Figure S5. 1D ^{1}H NMR spectrum of **2a** in $CDCl_3$ at T = 303……S5

Figure S6. 1D ^{13}C NMR spectrum of **2a** in $CDCl_3$ at T = 303……S5

Figure S7. MALDI-TOF mass spectrum of **2a**……S6

Figure S8. 1D ^{1}H NMR spectrum of **2b** in $CDCl_3$ at T = 303……S7

Figure S9. 1D ^{13}C NMR spectrum of **2b** in $CDCl_3$ at T = 303……S7

Figure S10. MALDI-TOF mass spectrum of **2b**……S8

Figure S11. 1D ^{1}H NMR spectrum of **2b** in $CDCl_3$ at T = 303……S9

Figure S12. 1D ^{13}C NMR spectrum of **2b** in $CDCl_3$ at T = 303……S9

Figure S13. MALDI-TOF mass spectrum of **2b**……S10

Figure S14. 1D ^{1}H NMR spectrum of **4a** in $CDCl_3$ at T = 303……S11

Figure S15. 1D ^{13}C NMR spectrum of **4a** in $CDCl_3$ at T = 303……S11

Figure S16. 1D ^{1}H NMR spectrum of **4b** in $CDCl_3$ at T = 303……S12

Figure S17. 1D ^{13}C NMR spectrum of **4b** in $CDCl_3$ at T = 303……S12

Figure S18. MALDI-TOF mass spectrum of **4b**……S13

Figure S19. 1D ^{1}H NMR spectrum of **12** in DMSO-d_6 at T = 303……S14

Figure S20. 1D ^{13}C NMR spectrum of **12** in DMSO-d$_6$ at T = 303……………………..S14
Figure S21. 1D ^{1}H NMR spectrum of **2b** in CDCl$_3$ at T = 303…………………………S15
Figure S22. 1D ^{13}C NMR spectrum of **2b** in CDCl$_3$ at T = 303…………………………S15
Figure S23. MALDI-TOF mass spectrum of **2b**…………………………………......S16
Figure S24. 1D ^{1}H NMR spectrum of **6** in CDCl$_3$ at T = 303……………………………S16
Comparison of docking results obtained with AutoDock 4.2.6 and AutoDock Vina 1.1.2 programs……………………………………………………………………S17
Table S1 Estimated binding energies of binding to hAChE obtained with AutoDock 4.2.6 and AutoDock Vina 1.1.2……………………………………………………………S18
Figure S25. Binding poses of compound **5a** inside hAChE obtained with Autodock 4.2.6 (carbon atoms are shown cyan), AutoDock Vina1.1.2…………………………………..S19
References…………………………………………………………………..……S19

NMR and MS study of the compounds synthesized

NMR experiments details

All NMR experiments were performed with 400 MHz for ^{1}H NMR and 100 MHz for ^{13}C NMR spectrometer equipped with 5 mm diameter gradient inverse broad band probehead and a pulsed gradient unit capable of producing magnetic field pulse gradients in the *z*-direction of 53.5 G•cm^{-1}. NMR experiments were carried out at 303 K. Chemical shifts (δ in ppm) were referenced to the solvents DMSO-d$_6$ (δ = 2.49 ppm for ^{1}H and 39.5 ppm for ^{13}C NMR), CDCl$_3$ (δ = 7.26 ppm for ^{1}H and 77.0 ppm for ^{13}C NMR).

MS experiments details

MALDI-TOF mass spectra were recorded in a positive and negative ion mode on a Bruker ULTRAFLEX III mass spectrometer (Bruker Daltonic GmbH, Bremen, Germany) using *p*-nitroaniline as a matrix for 10^{-3} mg/ml solutions in MeOH. A Nd:YAG laser (λ = 355 nm, repetition rate 100 Hz) was used. The mass spectrum was obtained with an accelerating voltage of 25 kV and an ion extraction delay time of 30 ns. The resulting mass spectrum was formed due to multiple laser irradiation of the crystal (50 shots). The metal target MTP AnchorChipTM was used. Portions (0.5 μl) of a 1% matrix solution in acetonitrile and sample solution were consecutively applied onto the target and evaporated. The polyethylene glycol was used to calibrate the mass scale of the device. The data was obtained using the FlexControl program and processed using the FlexAnalysis 3.0 program (Bruker Daltonik GmbH, Germany).

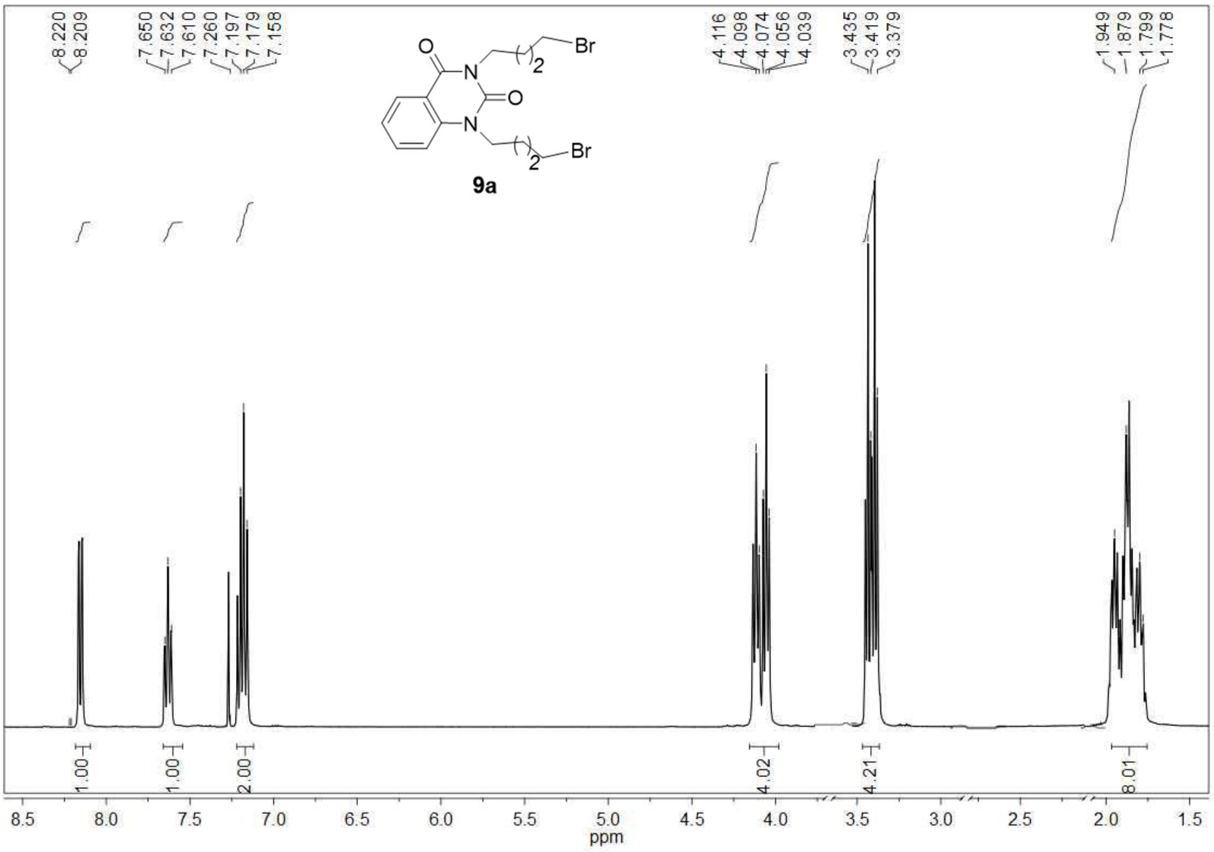

Figure S1. 1D ^{1}H NMR spectrum of **9a** in $CDCl_3$ at T = 303

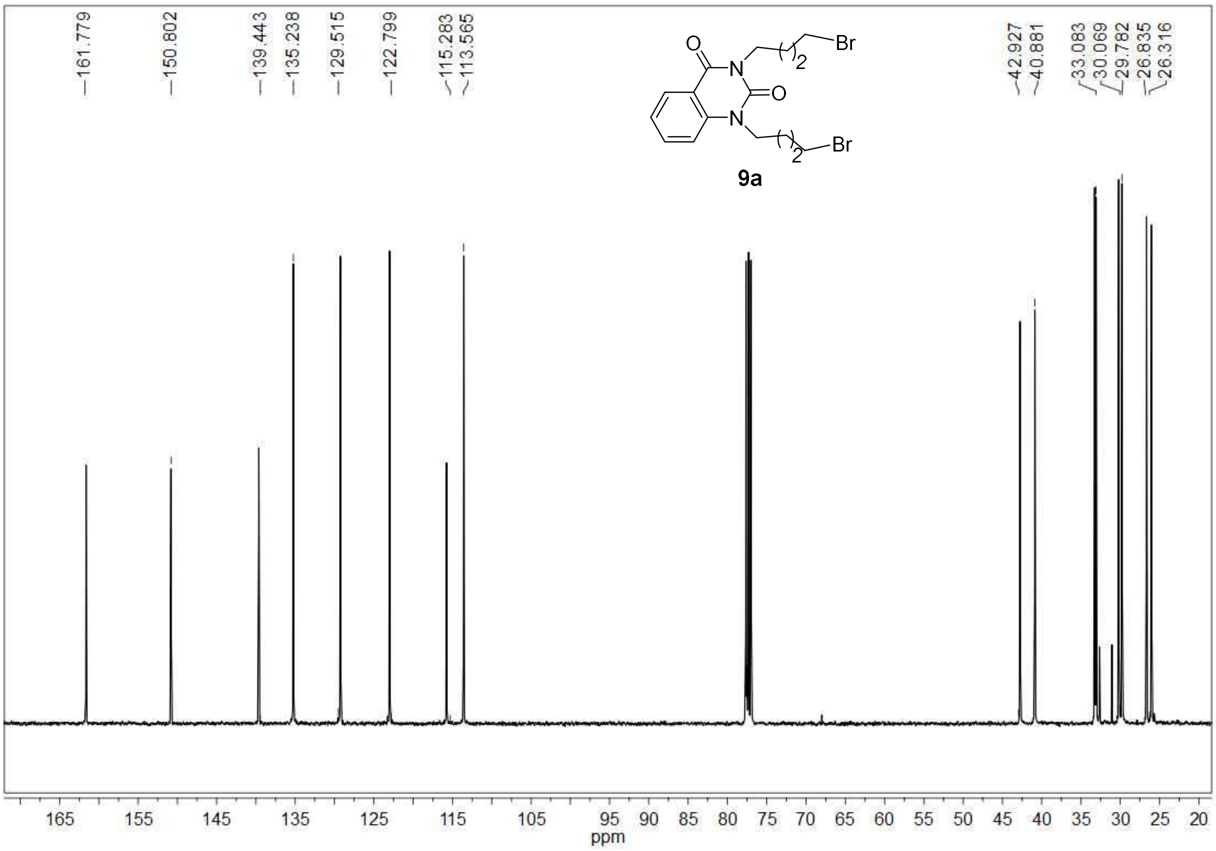

Figure S2. 1D ^{13}C NMR spectrum of **9a** in $CDCl_3$ at T = 303

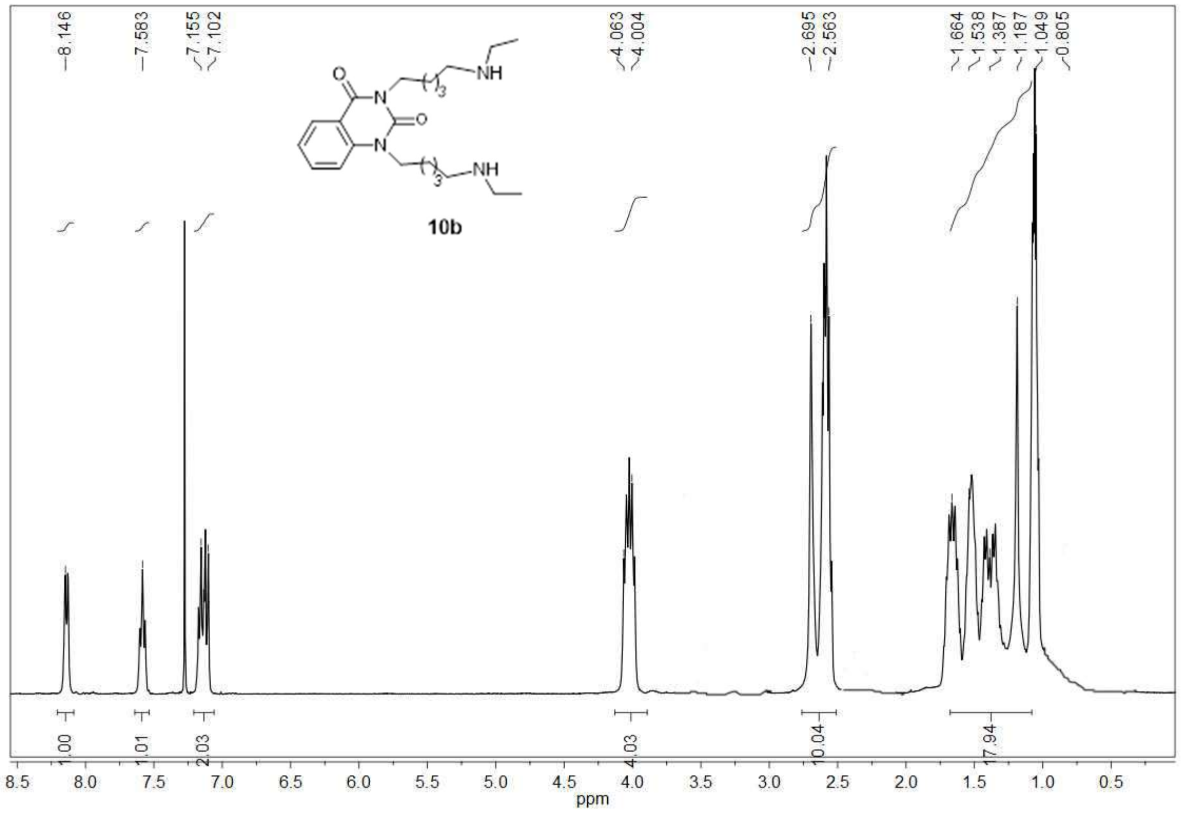

Figure S3. 1D ^{1}H NMR spectrum of **10b** in $CDCl_3$ at T = 303

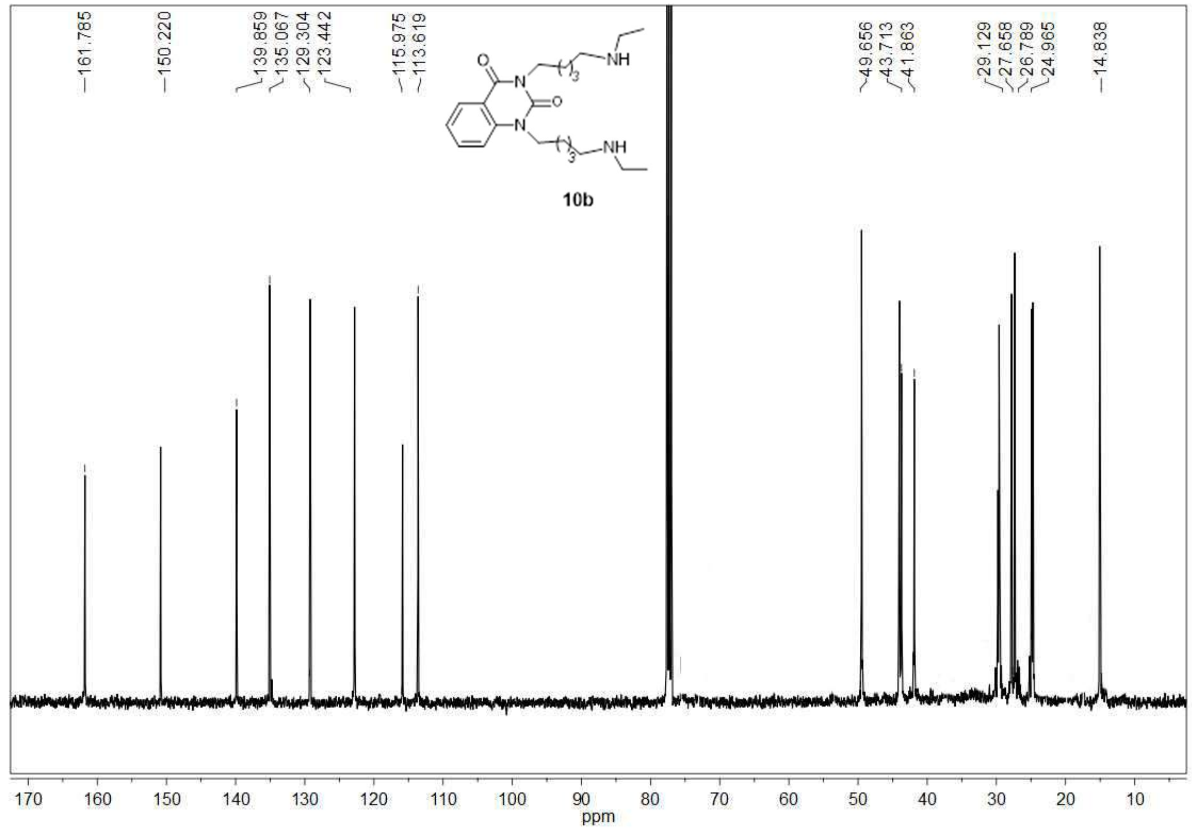

Figure S4. 1D ^{13}C NMR spectrum of **10b** in $CDCl_3$ at T = 303

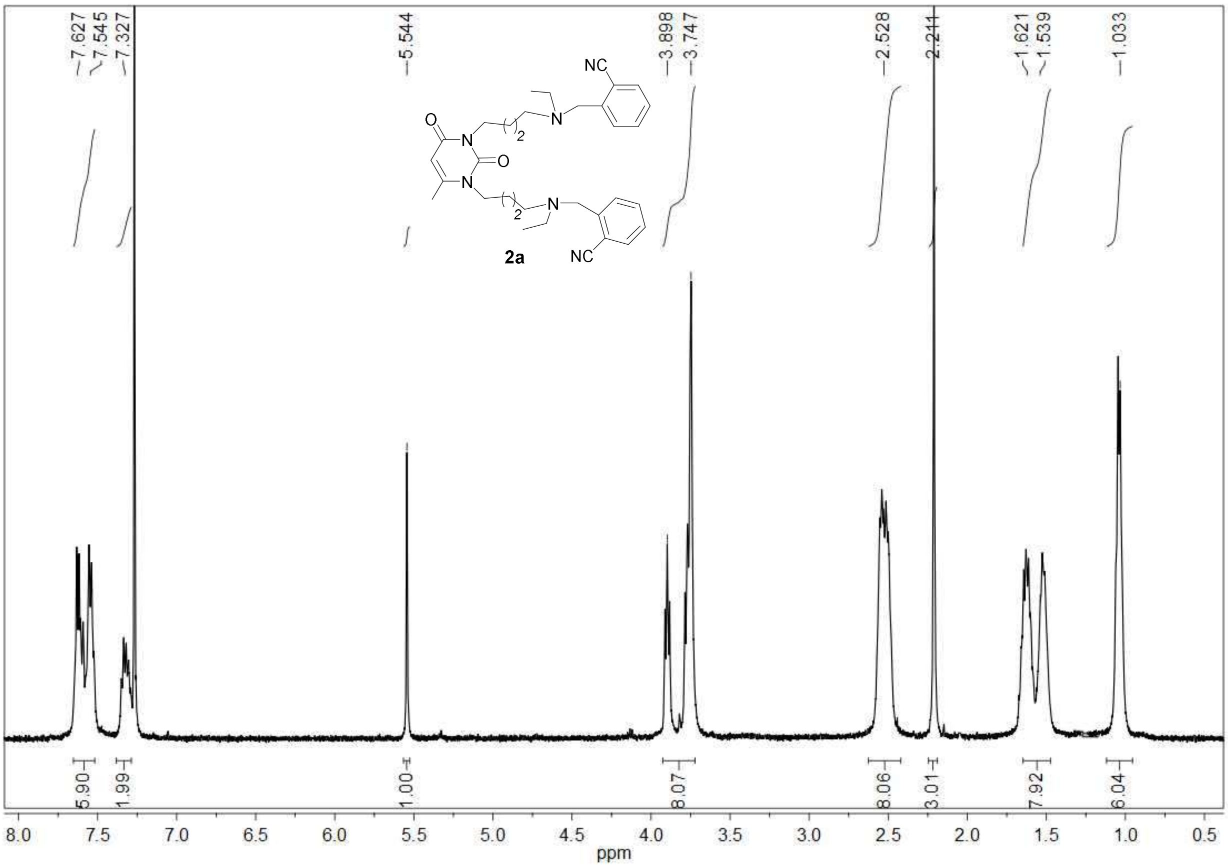

Figure S5. 1D ^{1}H NMR spectrum of **2a** in $CDCl_3$ at T = 303

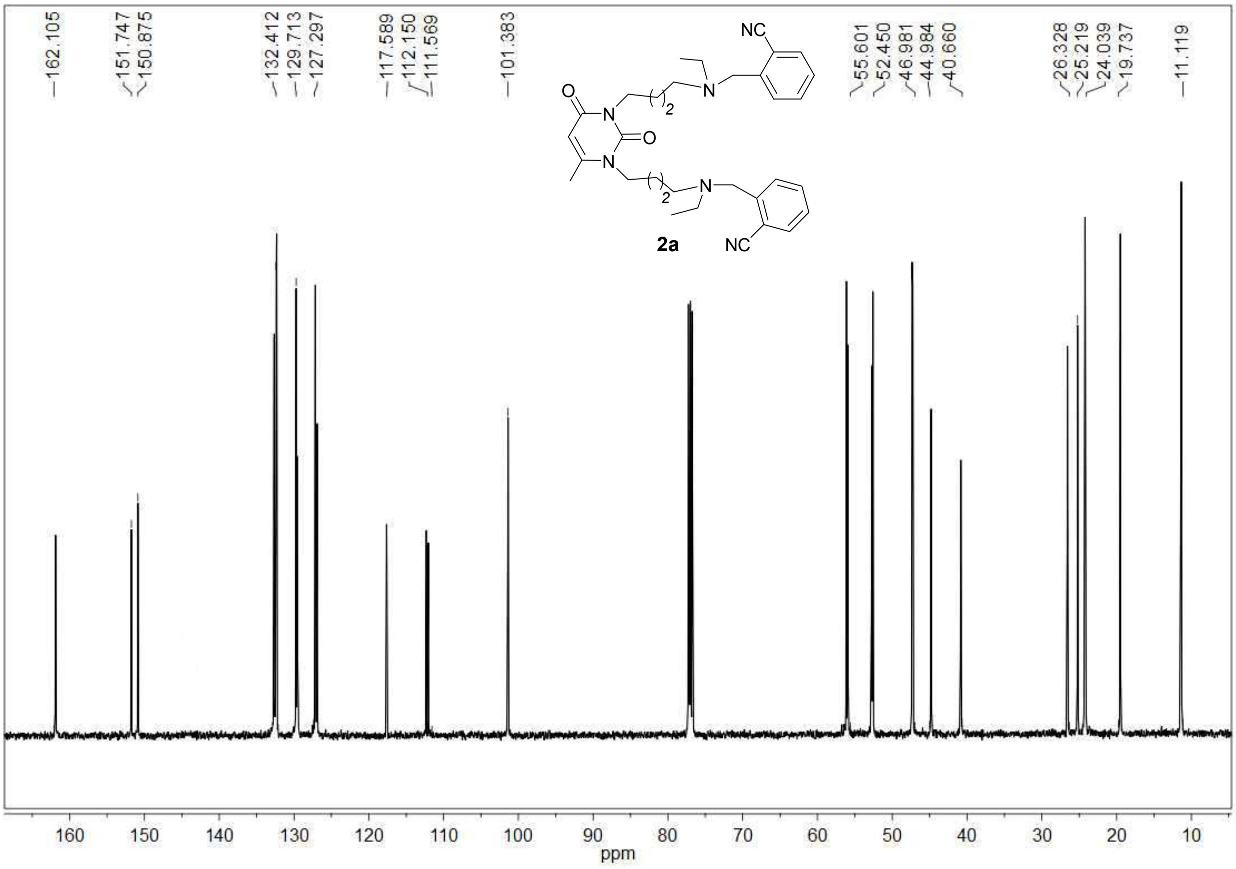

Figure S6. 1D ^{13}C NMR spectrum of **2a** in $CDCl_3$ at T = 303

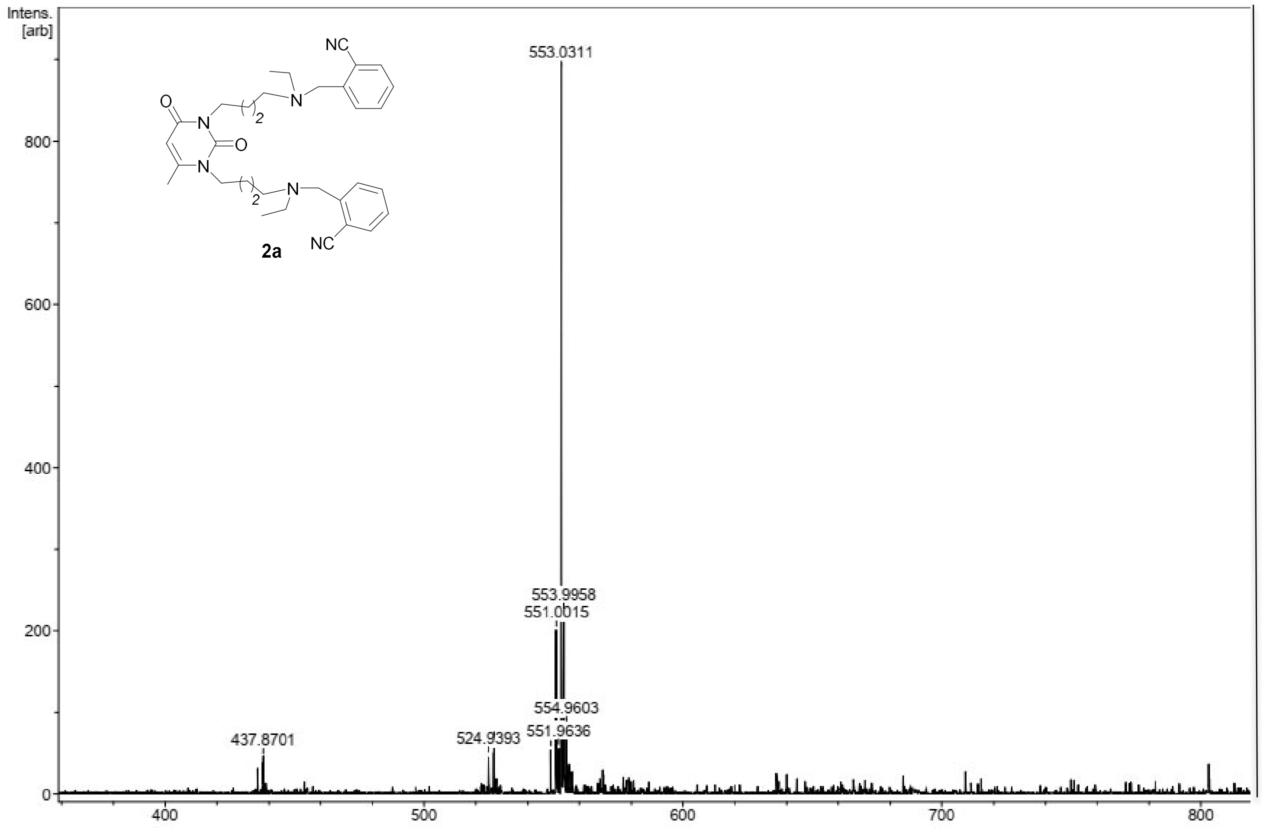

Figure S7. MALDI-TOF mass spectrum of **2a**

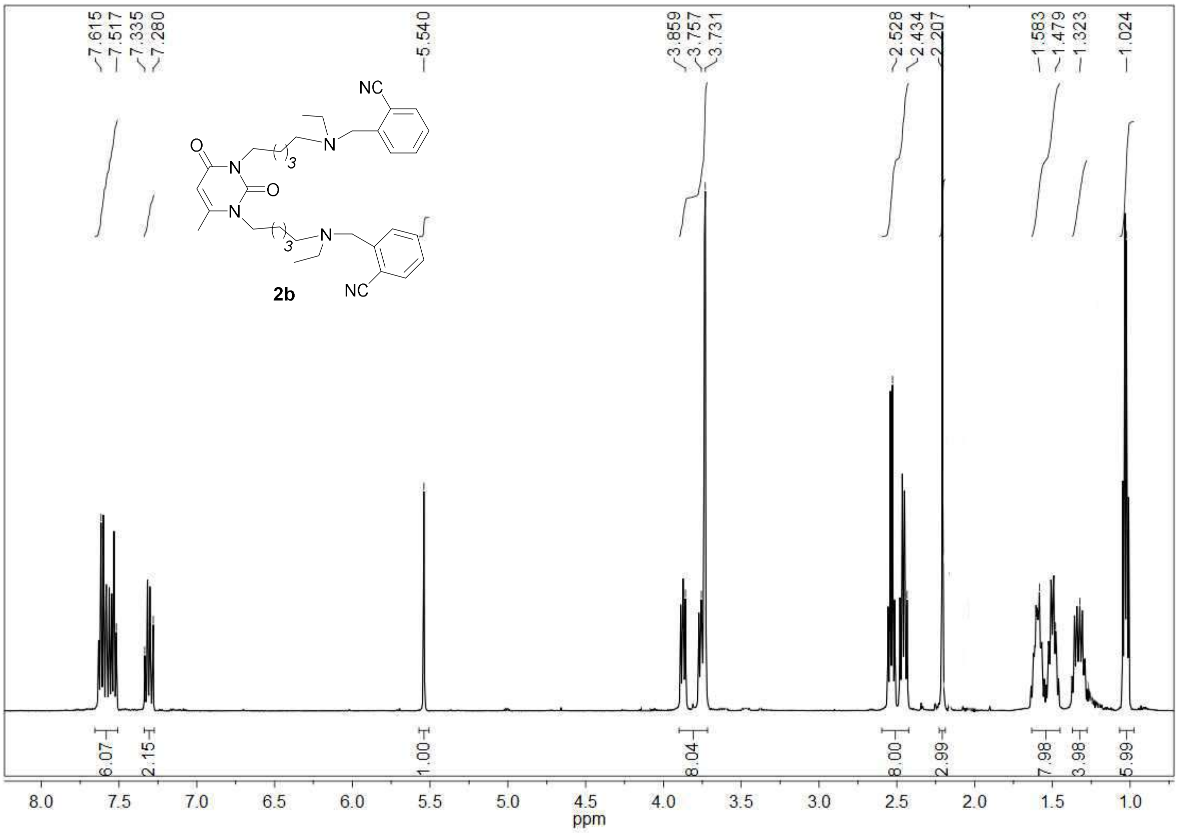

Figure S8. 1D ^{1}H NMR spectrum of **2b** in $CDCl_3$ at T = 303

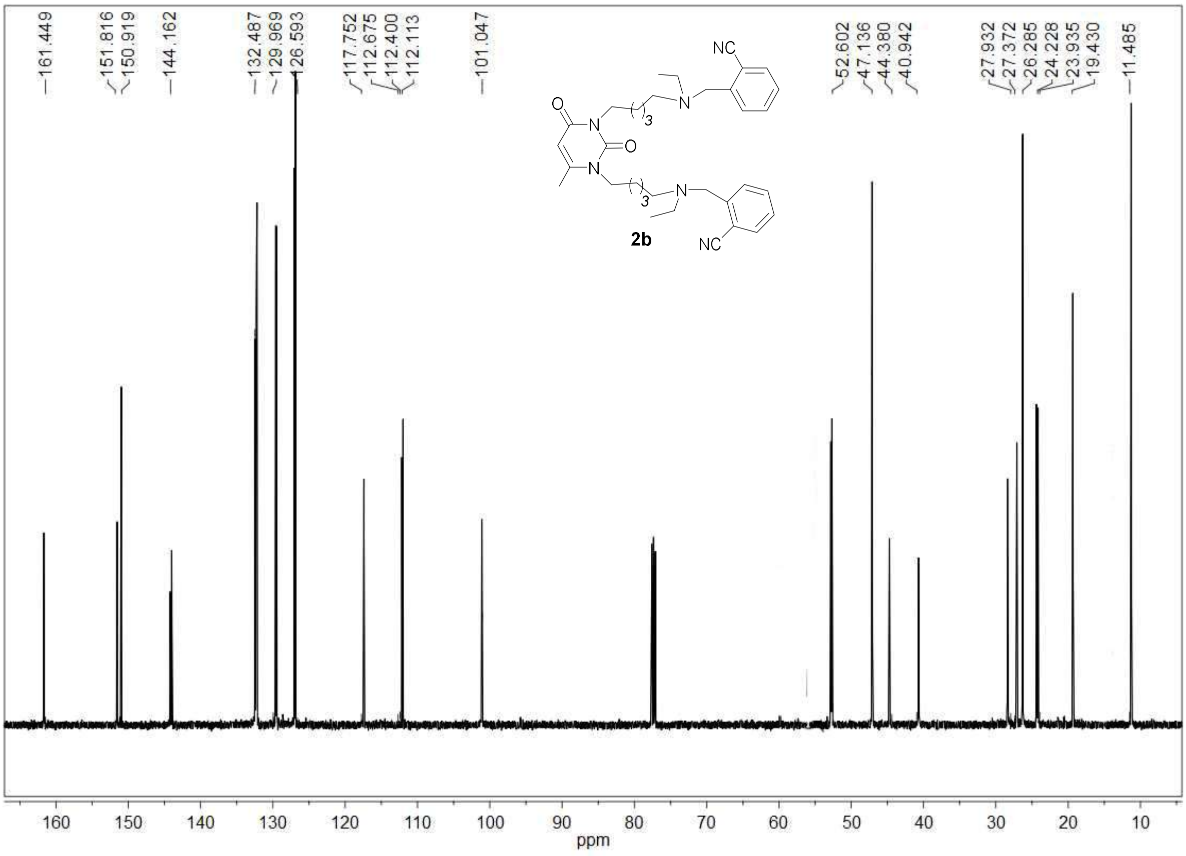

Figure S9. 1D ^{13}C NMR spectrum of **2b** in $CDCl_3$ at T = 303

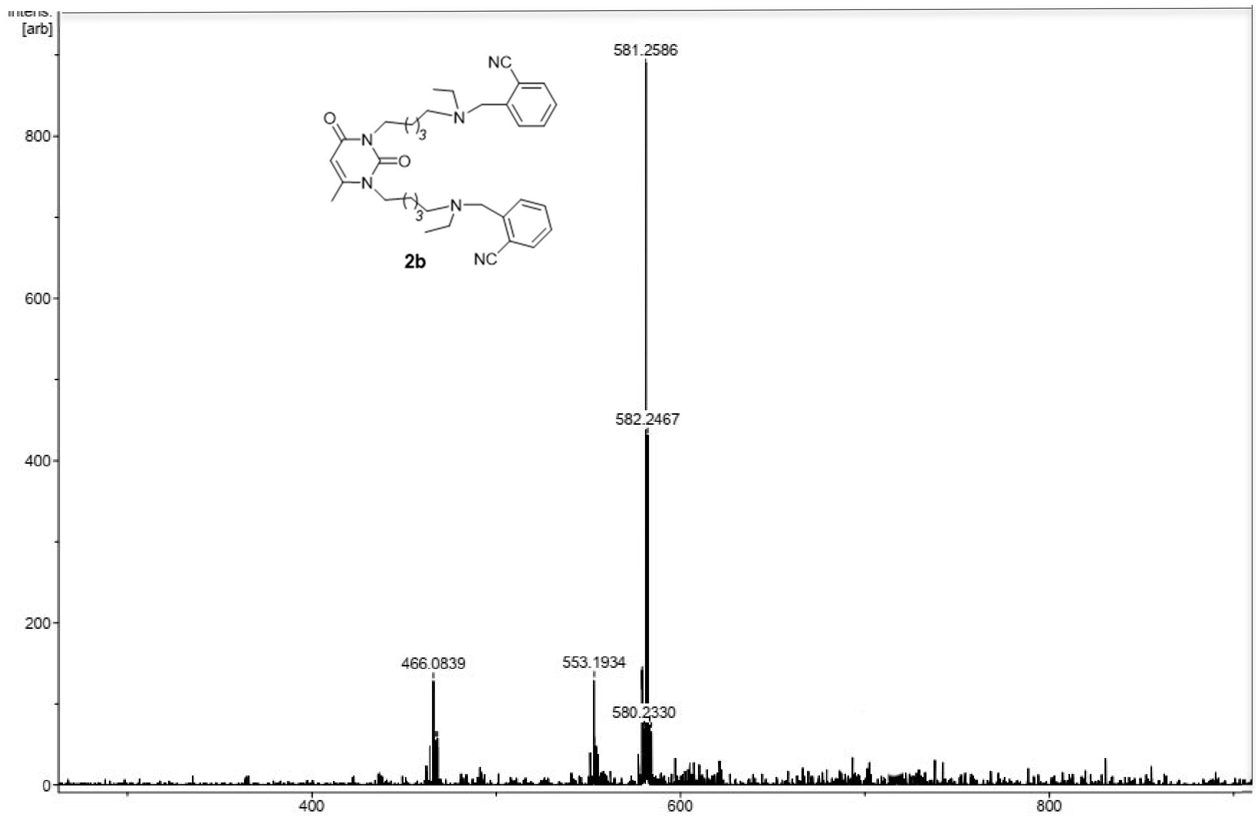

Figure S10. MALDI-TOF mass spectrum of **2b**

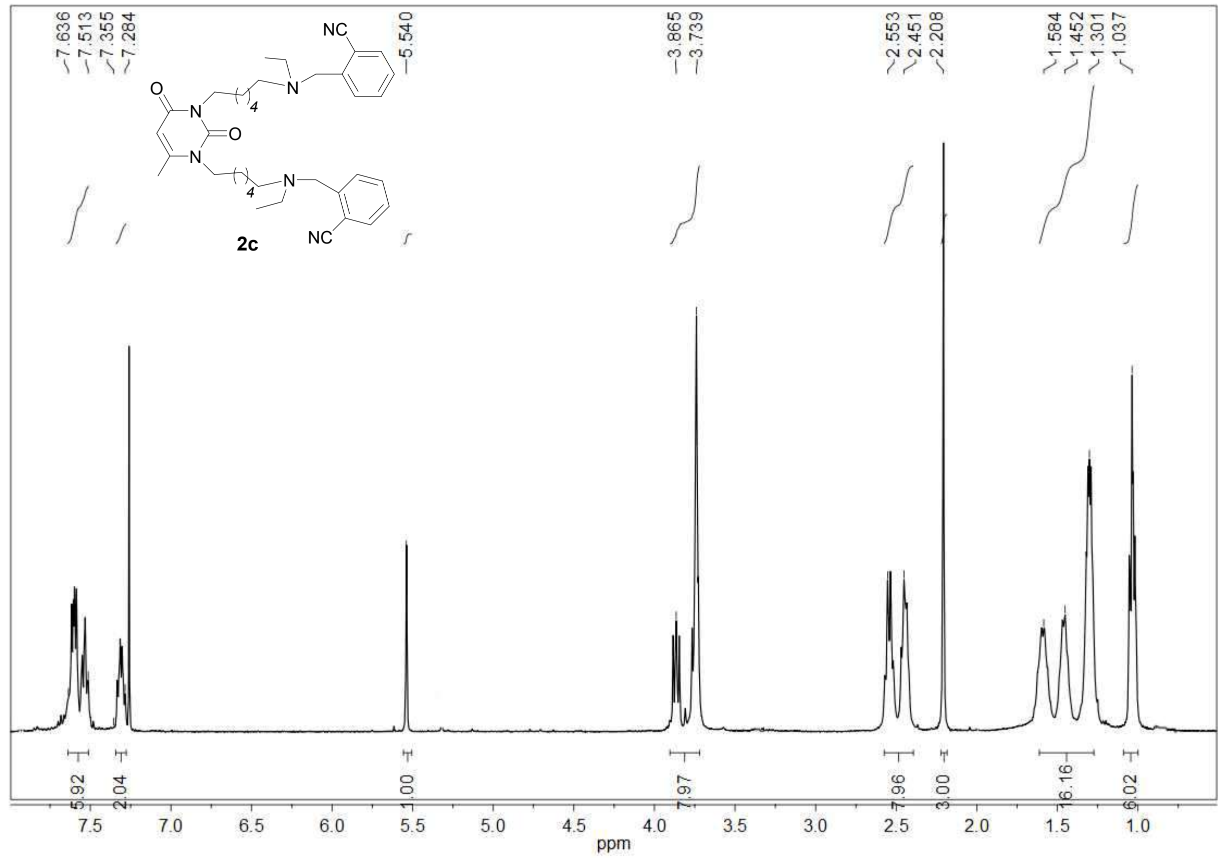

Figure S11. 1D ^{1}H NMR spectrum of **2c** in $CDCl_3$ at T = 303

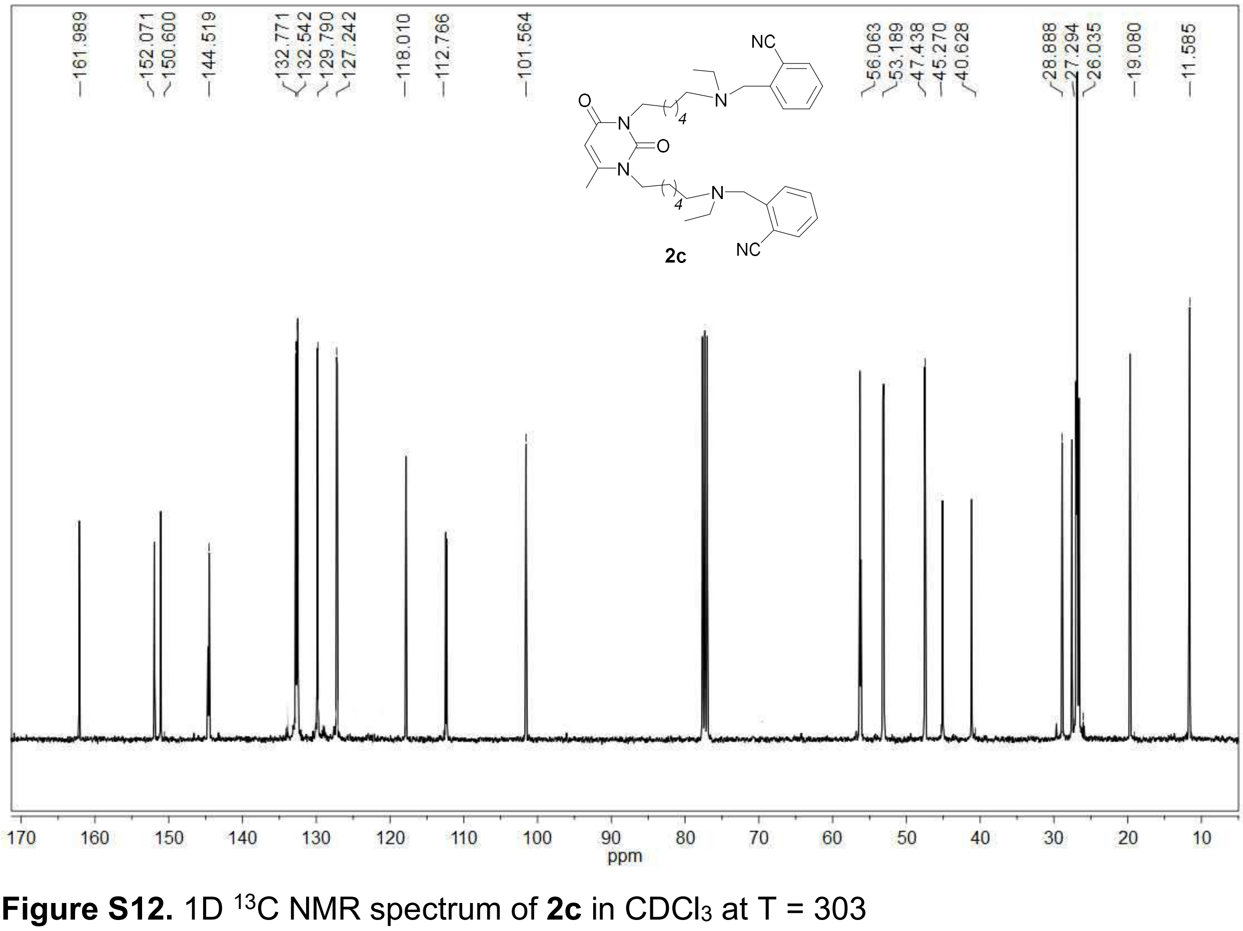

Figure S12. 1D ^{13}C NMR spectrum of **2c** in $CDCl_3$ at T = 303

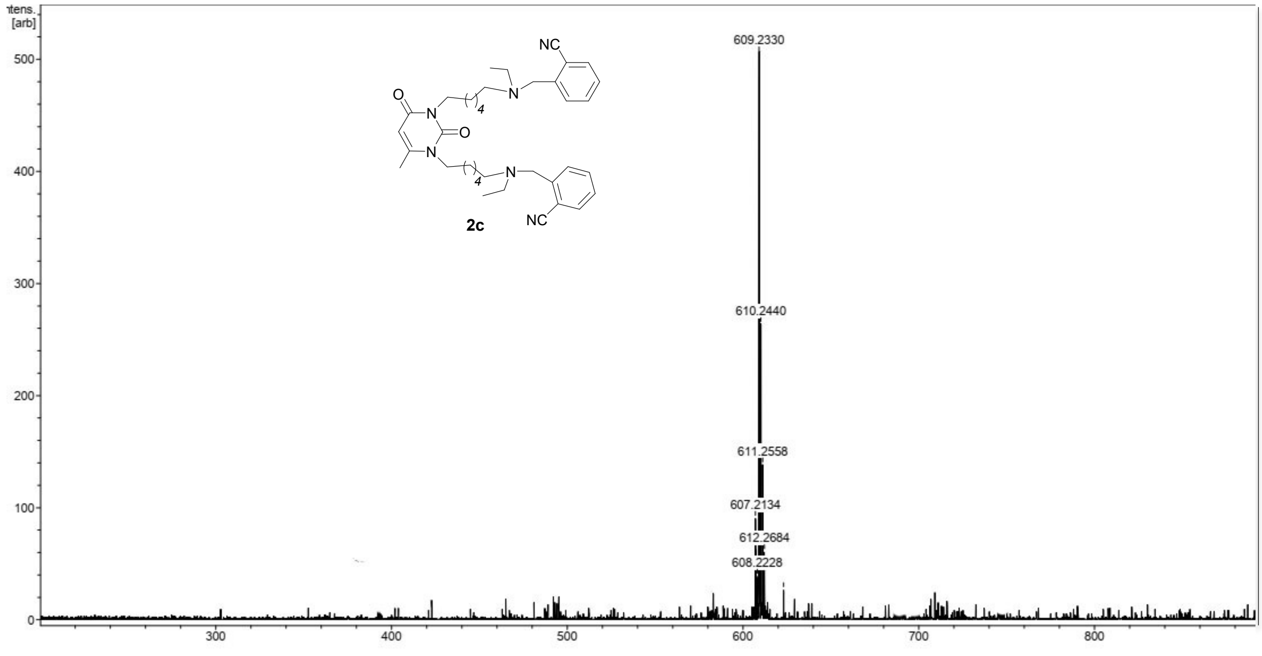

Figure S13. MALDI-TOF mass spectrum of **2c**

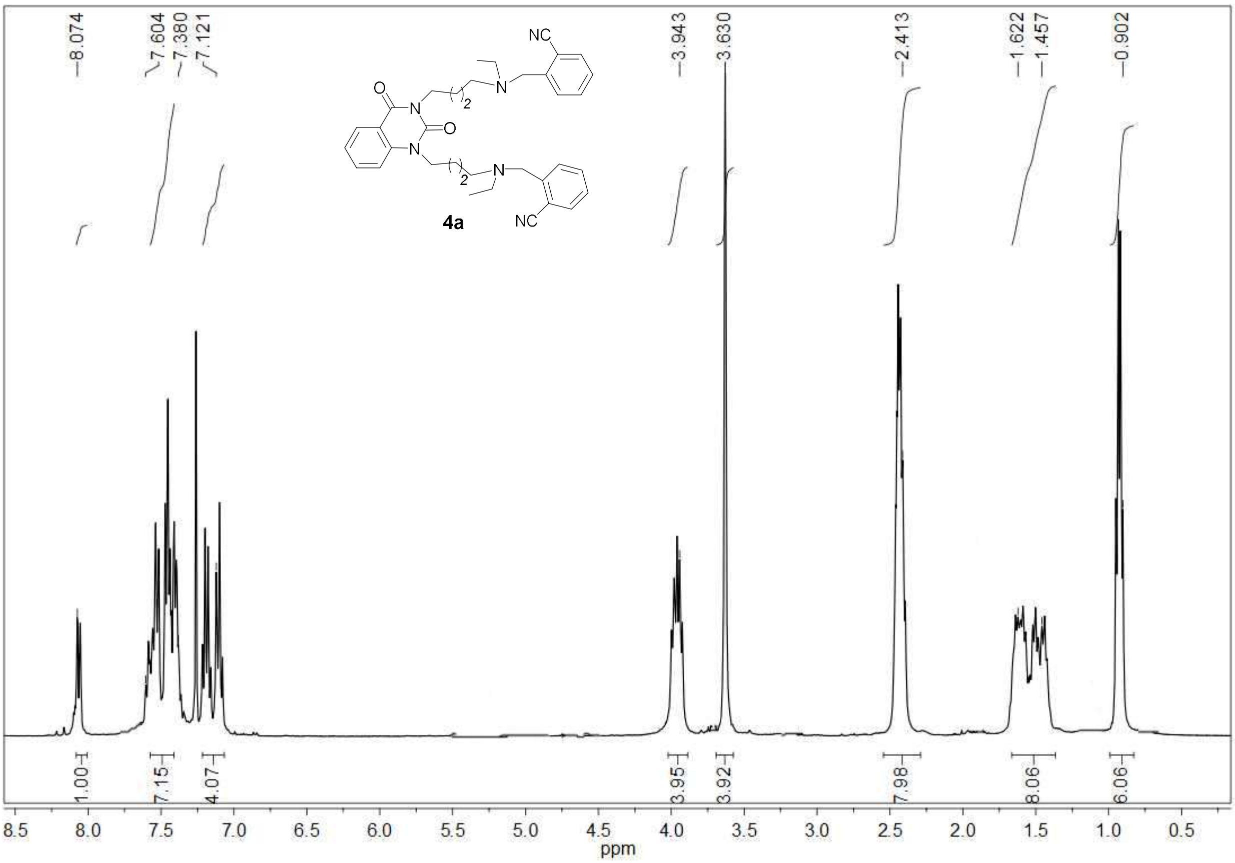

Figure S14. 1D ^{1}H NMR spectrum of **4a** in $CDCl_3$ at T = 303

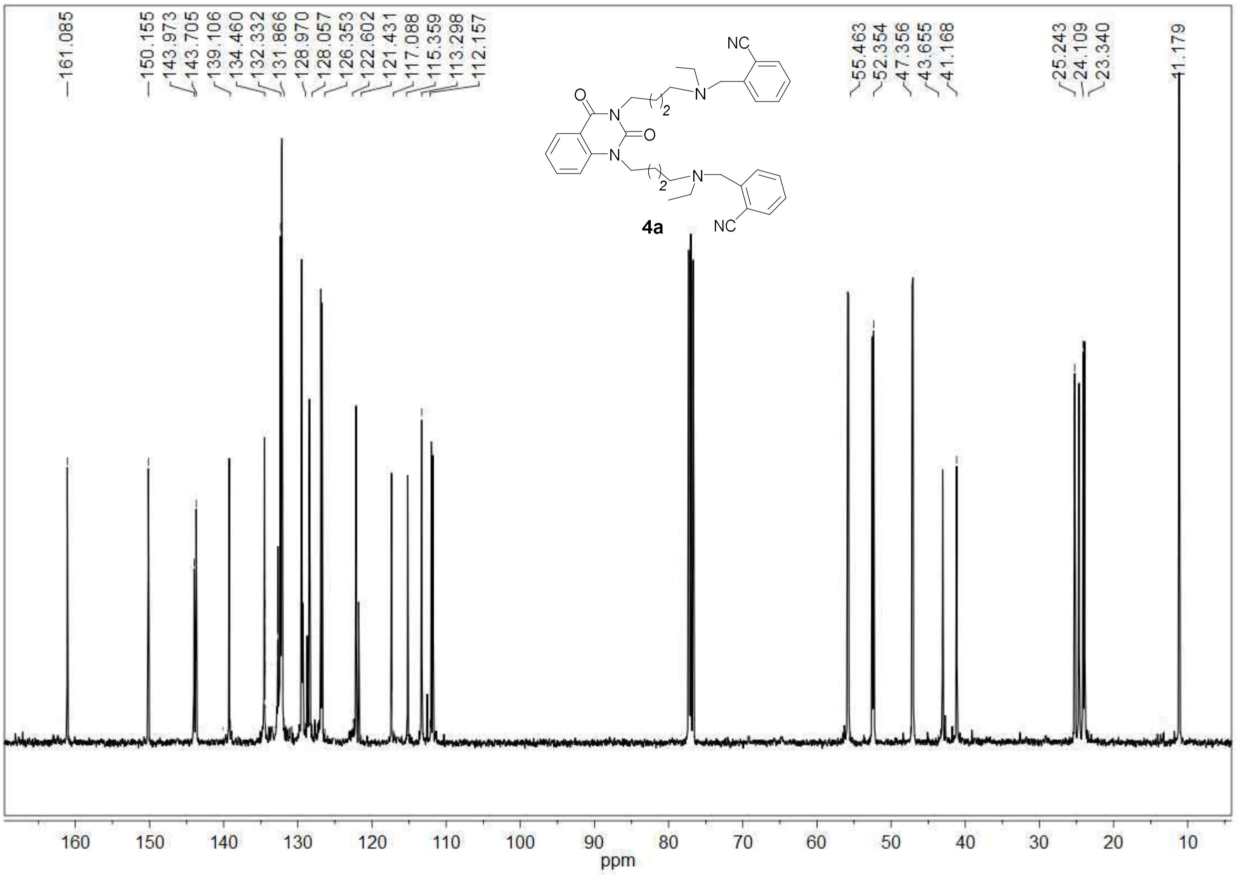

Figure S15. 1D ^{13}C NMR spectrum of **4a** in $CDCl_3$ at T = 303

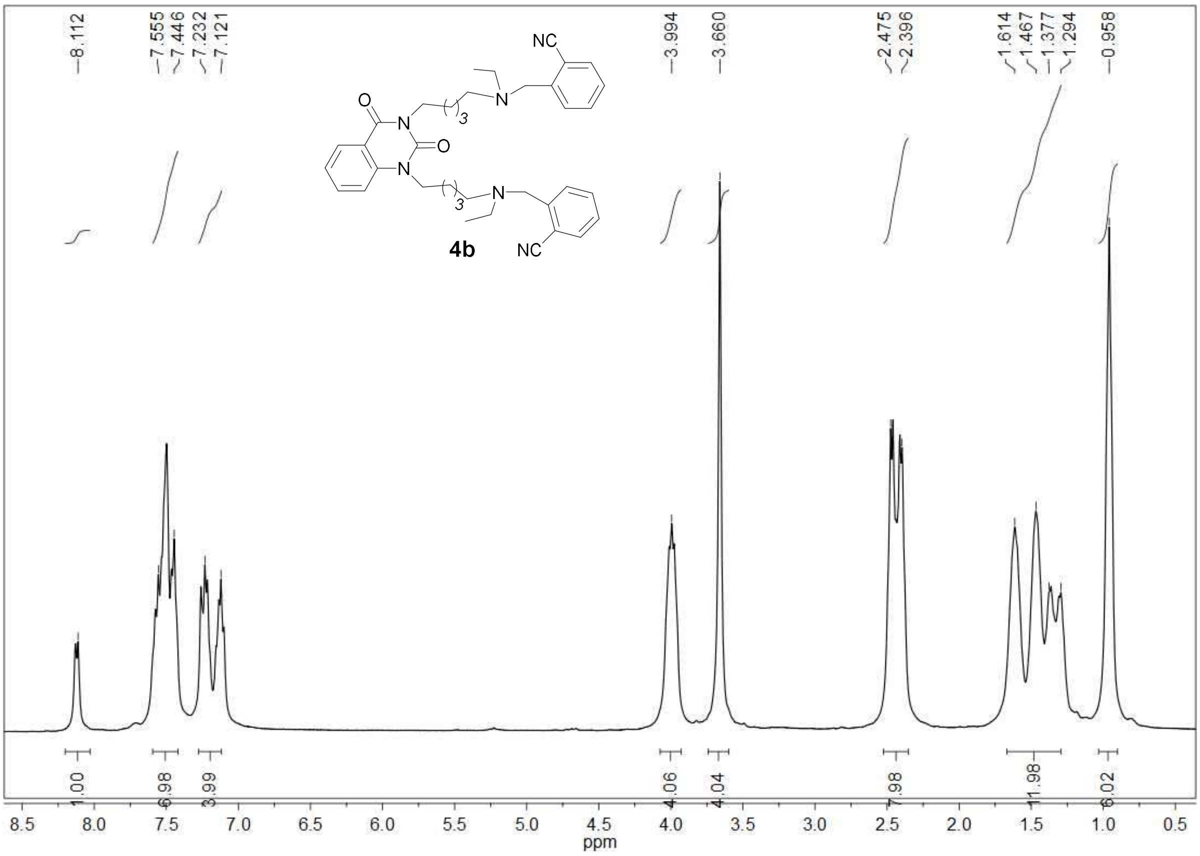

Figure S16. 1D ^{1}H NMR spectrum of **4b** in $CDCl_3$ at T = 303

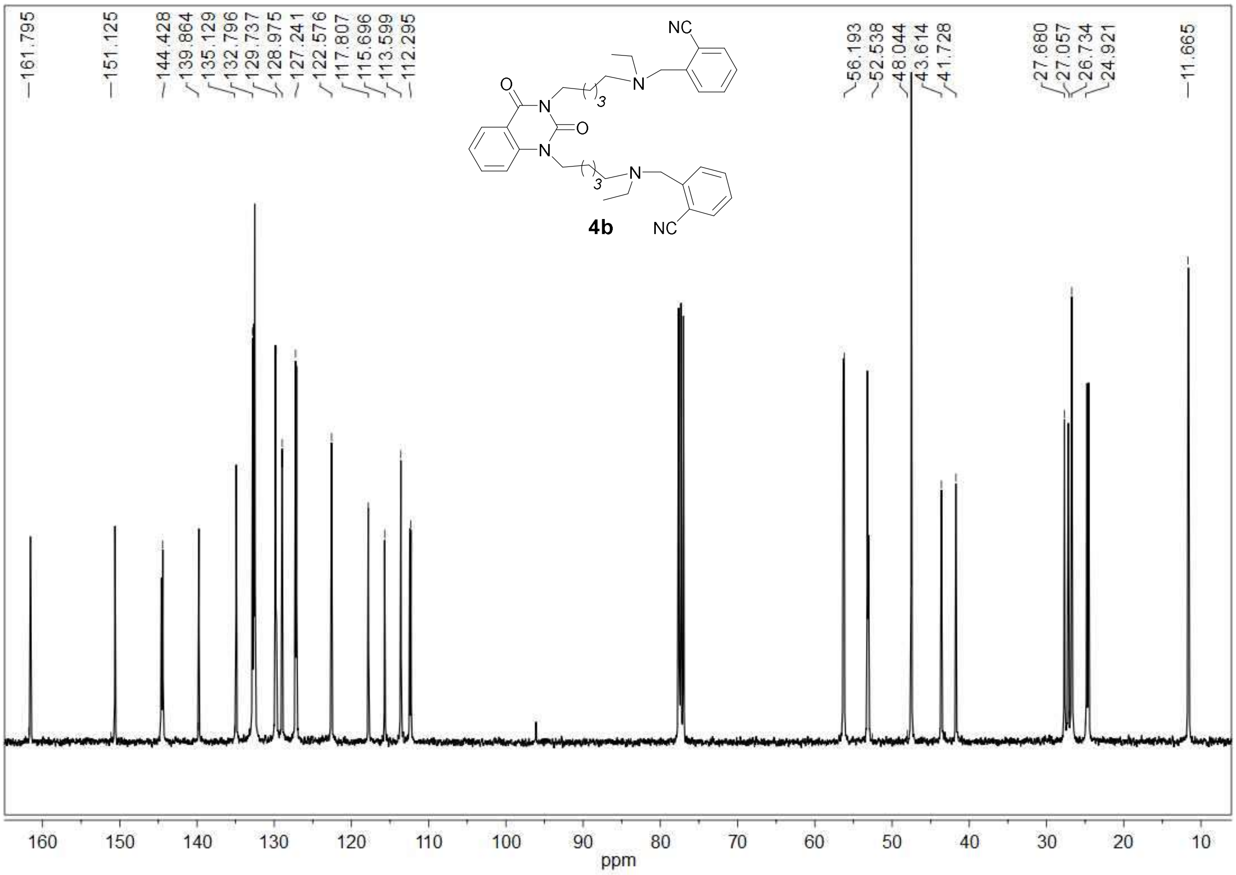

Figure S17. 1D ^{13}C NMR spectrum of **4b** in $CDCl_3$ at T = 303

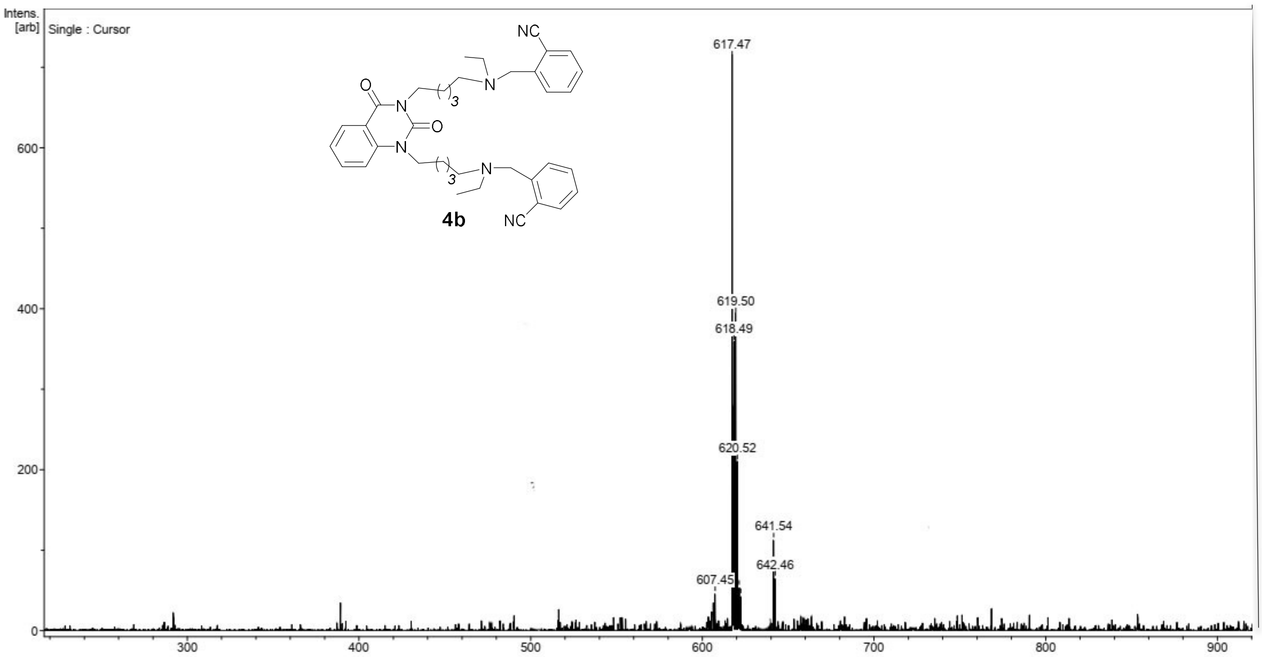

Figure S18. MALDI-TOF mass spectrum of **4b**

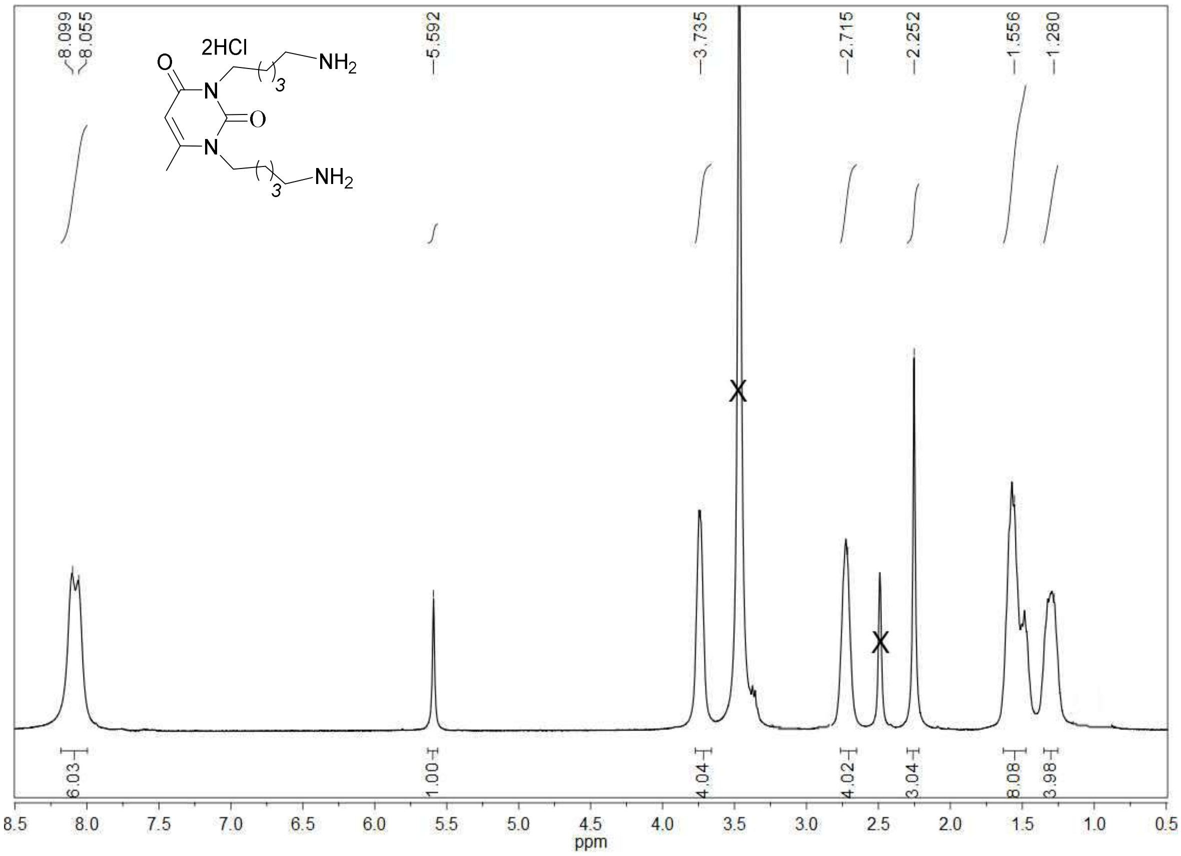

Figure S19. 1D ^{1}H NMR spectrum of **12** in DMSO-d_6 at T = 303, x - residual solvent peaks (H_2O, DMSO)

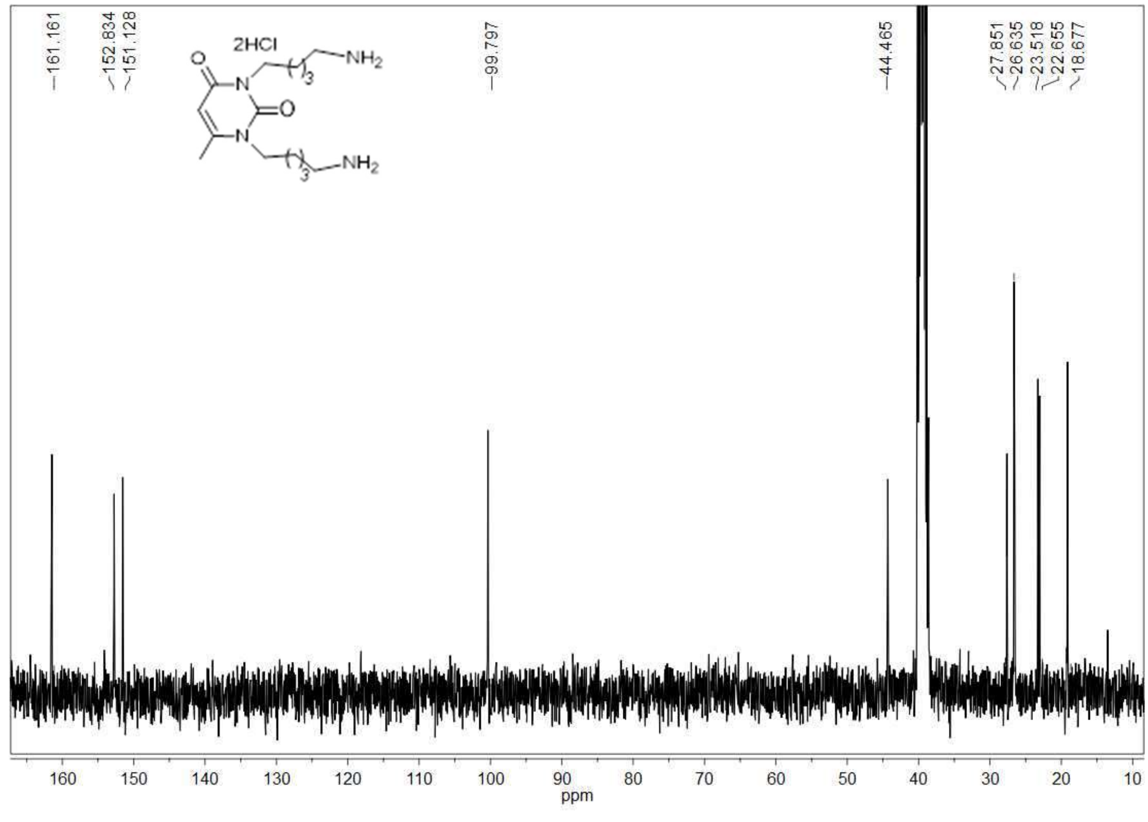

Figure S20. 1D ^{13}H NMR spectrum of **12** in DMSO-d_6 at T = 303

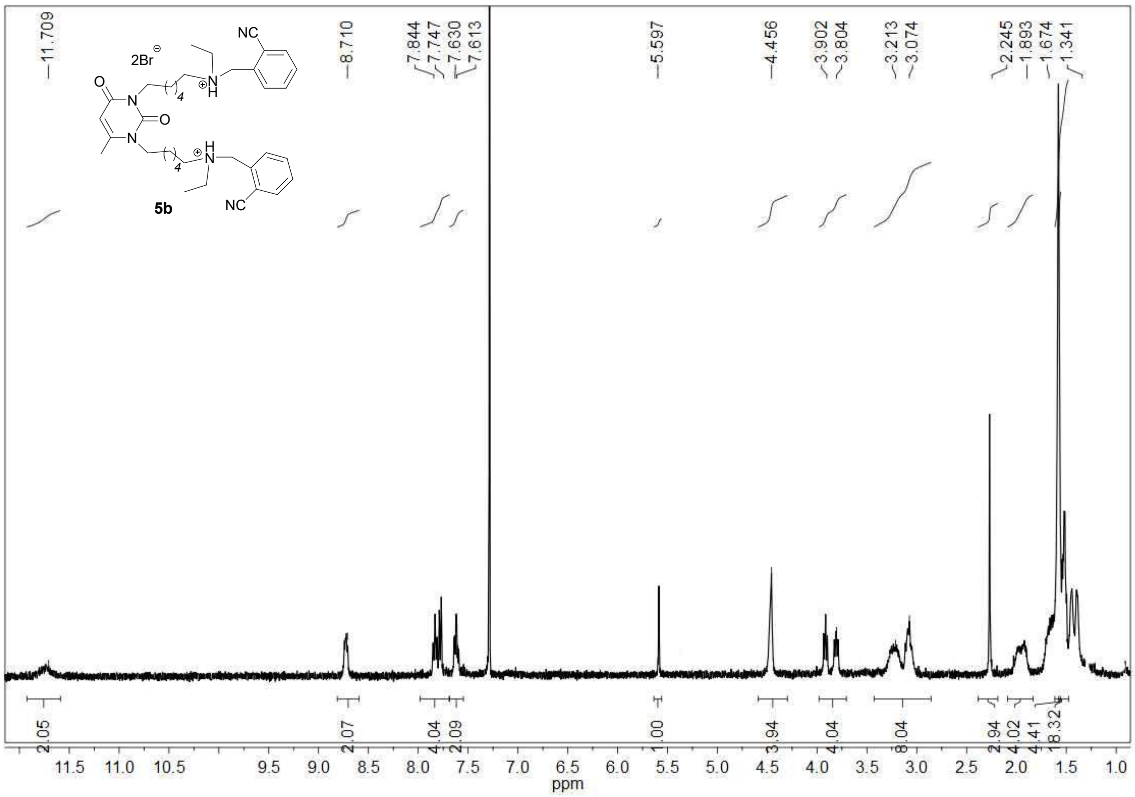

Figure S21. 1D ^{1}H NMR spectrum of **5b** in $CDCl_3$ at T = 303

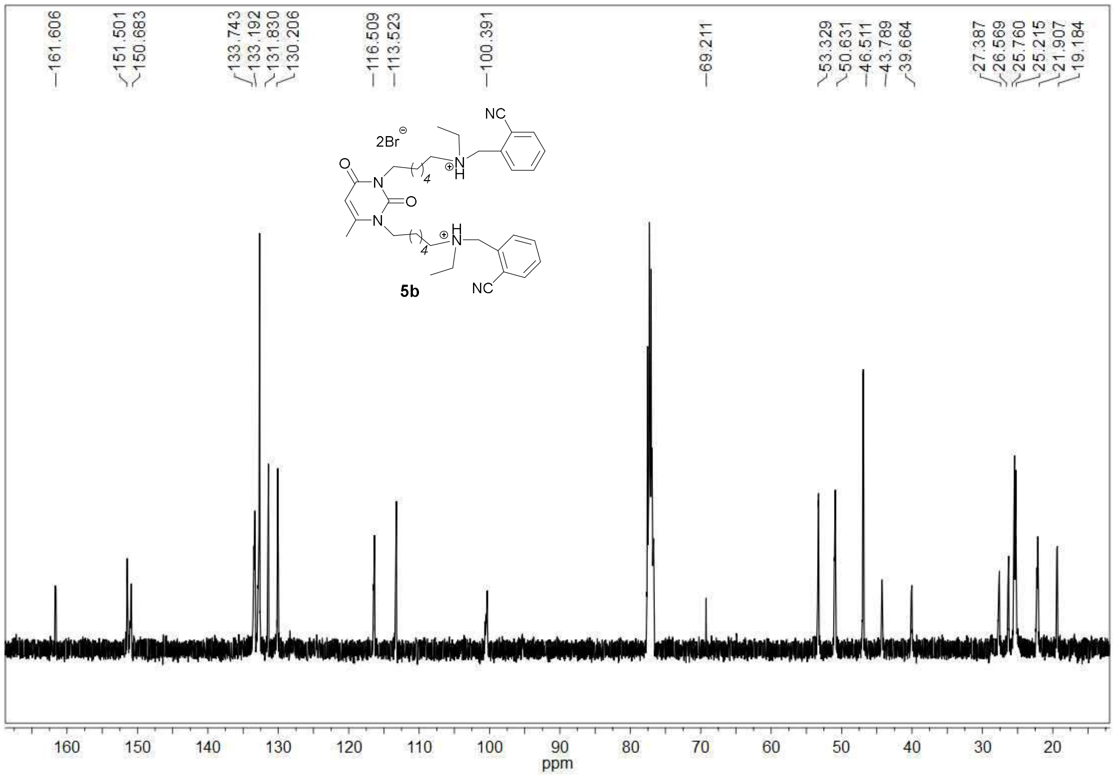

Figure S22. 1D ^{13}C NMR spectrum of **5b** in $CDCl_3$ at T = 303

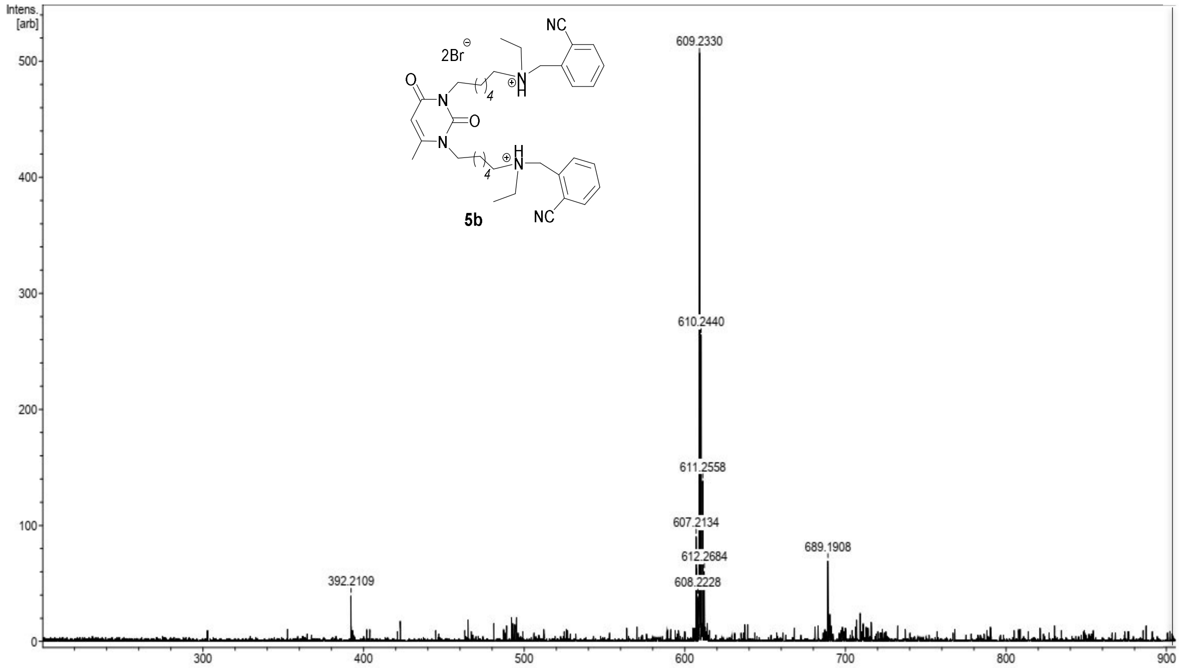

Figure S23. MALDI-TOF mass spectrum of **5b**

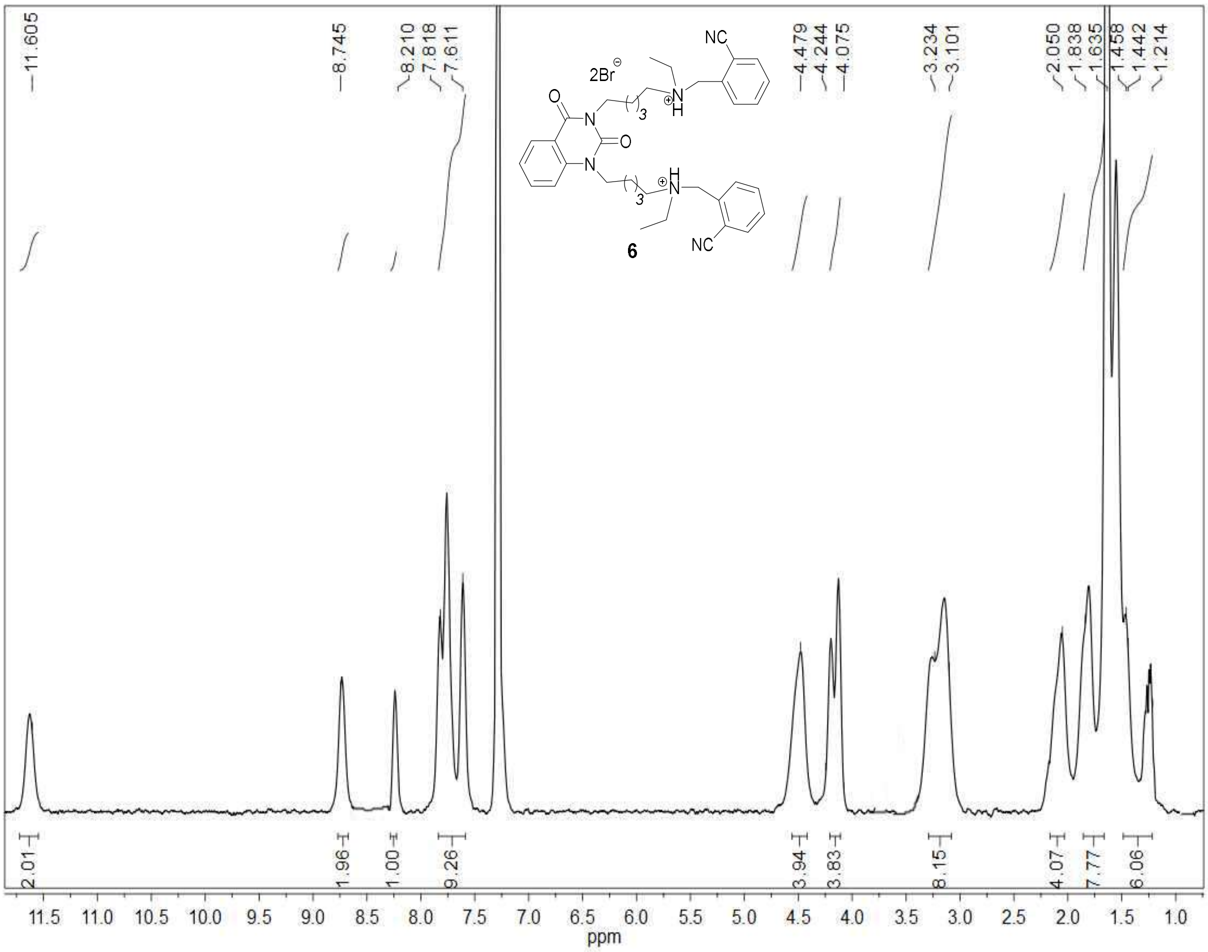

Figure S24. 1D ^{1}H NMR spectrum of **6** in $CDCl_3$ at T = 303

Comparison of docking results obtained with AutoDock 4.2.6 and AutoDock Vina 1.1.2 programs

There are many studies comparing AutoDock and AutoDock Vina with each other and other docking programs published [1-4]. They mostly report comparable accuracy, or even better results with Vina. However, most of test sets had relatively small compounds. In a work, dealing with big molecules, both programs demonstrated very poor results with standard docking parameters [4]. In the current study, we deal with relatively large molecules with many torsional degrees of freedom (18-22). To deal with such molecules, Autodock Vina allows to change only *exhaustiveness* value (default 8, maximum 1000), while AutoDock 4.2.6 allows to modify many Lamarckian Genetic Algorithm parameters. The program developers recommend to increase *number of evaluations*, *number of generations* and *population size* (http://autodock.scripps.edu/faqs-help/faq/which-values-of-the-genetic-algorithm-parameters-do-you-normally-use). Here, we compared results obtained by AutoDock 4.2.6 with increased docking parameters (were 256 *runs*, 25×10^6 *evaluations*, 27×10^4 *generations* and a *population size* of 3000), and Autodock Vina with *exhaustiveness* parameter set to 40 and 1000.

Obtained results show, that in the present case AutoDock finds better binding poses, than Vina. This is reflected both in estimated binding energies (Table S1), and binding poses (Figure S1). The main differences in the binding poses, influencing binding energy are shown for compound **5a** (Figure S1): (1) The cyanobenzyl ring located in the active site near the catalytic triad has π-π stacking interactions with Trp86 indole ring in case of AutoDock results, while in case of Vina results it is moved aside from Trp86 indole ring, what impairs π-π stacking. In case of Vina results, with *exhaustiveness* parameter set to 1000, this is partly compensated by formation of hydrogen bond between the cyano-group and Tyr124 side chain, though it is 2.6Å long, and thus it is rather weak; (2) hydrogen bond between oxygen atom of the 6-methyluracil ring and Phe295 main chain amide group was found only by AutoDock, while in binding pose obtained with Vina (*exhaustiveness* 1000) 6-methyluracil ring oxygen atom is located further from the NH group, and with *exhaustiveness* parameter set to 40 6-methyluracil ring is flipped, and methyl group is located in this area; (3) hydrogen bond between the

second cyanobenzyl ring cyano-group and S293 main chain amide group[1] was found by both AutoDock and Vina (*exhaustiveness* 1000), but not Vina (*exhaustiveness* 40).

Vina is much faster and easier to set up than AutoDock. This is particularly useful for high throughput screening of large ligand libraries. However, in case of ligands with many torsional degrees of freedom it should be used with cation. AutoDock 4.2.6 allows to increase various Lamarckian Genetic Algorithm parameters parameters, and in the present case this lead to better binding poses with more specific interactions than found with AutoDock Vina with maximum *exhaustiveness* parameter. Though, this is achieved by computational cost, ~36 processor hours for Vina *vs* ~60 processor hours for AutoDock.

Table S1. Estimated binding energies of binding to hAChE obtained with AutoDock 4.2.6 and AutoDock Vina 1.1.2, kcal/mol.

	AutoDock 4.2.6	Vina	
		exhaustiveness 40	exhaustiveness 1000
**2a**	-9.92	-9.4	-9.5
**2b**	-9.76	-9.4	-9
**2c**	-9.97	-9.1	-9.1
**4a**	-11.01	-10.5	-10.4
**4b**	-10.98	-10.2	-10.1
**5a**	-11.32	-8.9	-9.5
**5b**	-10.99	-9.4	-9.4
**6**	-12.38	-10.2	-10.2
**3**	-9.83	-9.2	-9.4

[1] The view angle presented in Figure S1 shows S293 side chain OH-group hydrogen atom lying on line connecting the ligand cyano-group nitrogen atom and S293 main chain NH-group hydrogen atom. This causes some confusion. In fact, hydrogen bond is between the ligand cyano-group nitrogen atom and S293 main chain NH-group hydrogen atom, see Figure 3,A in the main text for another view angle.

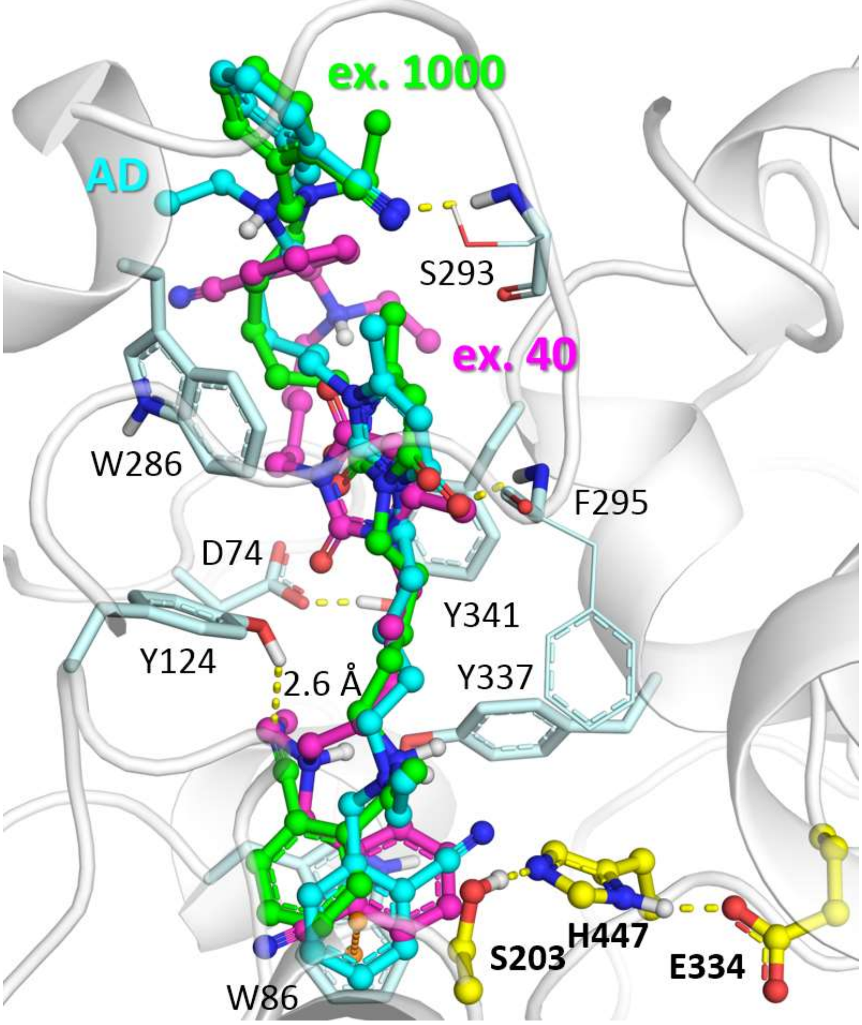

Figure S25. Binding poses of compound **5a** inside hAChE obtained with Autodock 4.2.6 (carbon atoms are shown cyan), AutoDock Vina (exhaustiveness parameter set to 40, carbon atoms are shown magenta, and exhaustiveness parameter set to 1000, carbon atoms are shown green). The catalytic triad is shown with yellow carbon atoms.

References

1. Gaillard, T. Evaluation of AutoDock and AutoDock Vina on the CASF-2013 Benchmark. *J. Chem. Inf. Model.* **2018**, *58*, 1697-1706, doi:10.1021/acs.jcim.8b00312.
2. Vieira, T.F.; Sousa, S.F. Comparing AutoDock and Vina in Ligand/Decoy Discrimination for Virtual Screening. *Applied Sciences* **2019**, *9*, 4538, doi:10.3390/app9214538.
3. Wang, Z.; Sun, H.; Yao, X.; Li, D.; Xu, L.; Li, Y.; Tian, S.; Hou, T. Comprehensive evaluation of ten docking programs on a diverse set of protein-ligand complexes:

the prediction accuracy of sampling power and scoring power. *Phys. Chem. Chem. Phys.* **2016**, *18*, 12964-12975, doi:10.1039/c6cp01555g.

4. Chang, M.W.; Ayeni, C.; Breuer, S.; Torbett, B.E. Virtual screening for HIV protease inhibitors: a comparison of AutoDock 4 and Vina. *PLoS One* **2010**, *5*, e11955, doi:10.1371/journal.pone.0011955.
